# Mucinous Cystadenocarcinoma Arising From a Mature Cystic Teratoma

**DOI:** 10.7759/cureus.84704

**Published:** 2025-05-23

**Authors:** B. Dina Rose, Divya Madhala, N Priyathersini

**Affiliations:** 1 Pathology, Sri Ramachandra Institute of Higher Education and Research, Chennai, IND

**Keywords:** ascites, cystic mass, malignant, mucinous cystadenocarcinoma, teratoma

## Abstract

Mucinous cystadenocarcinoma arising from mature cystic teratoma is an uncommon entity. The possibilities are either due to malignant transformation of benign teratoma into adenocarcinoma or a collision tumor between a mature cystic teratoma and a mucinous tumor. Here we present a case of a woman with abdominal distension and vague abdominal pain. Ultrasound abdomen showed a huge heterogeneous space-occupying lesion extending from the pelvis to the epigastrium with non-visualisation of the ovary, suggesting an ovarian origin. Further characterisation by contrast-enhanced computed tomography confirmed a hemorrhagic abdominopelvic multiseptate cystic lesion along with massive ascites. After surgery, the histopathological examination revealed mucinous cystadenocarcinoma arising from the mature cystic teratoma.

## Introduction

Mature teratomas are benign neoplasms, derived from at least two of the three germ cell layers. The incidence of malignant transformation of teratoma is 1-2% [1,2], most commonly squamous cell carcinoma. The occurrence of adenocarcinoma is rare. There are two hypotheses, including the presence of a collision tumor or malignant transformation occurring in a benign component, that need to be considered. Here we report the case of a 52-year-old female with mucinous cystadenocarcinoma arising from mature cystic teratoma.

## Case presentation

**Figure 1:**
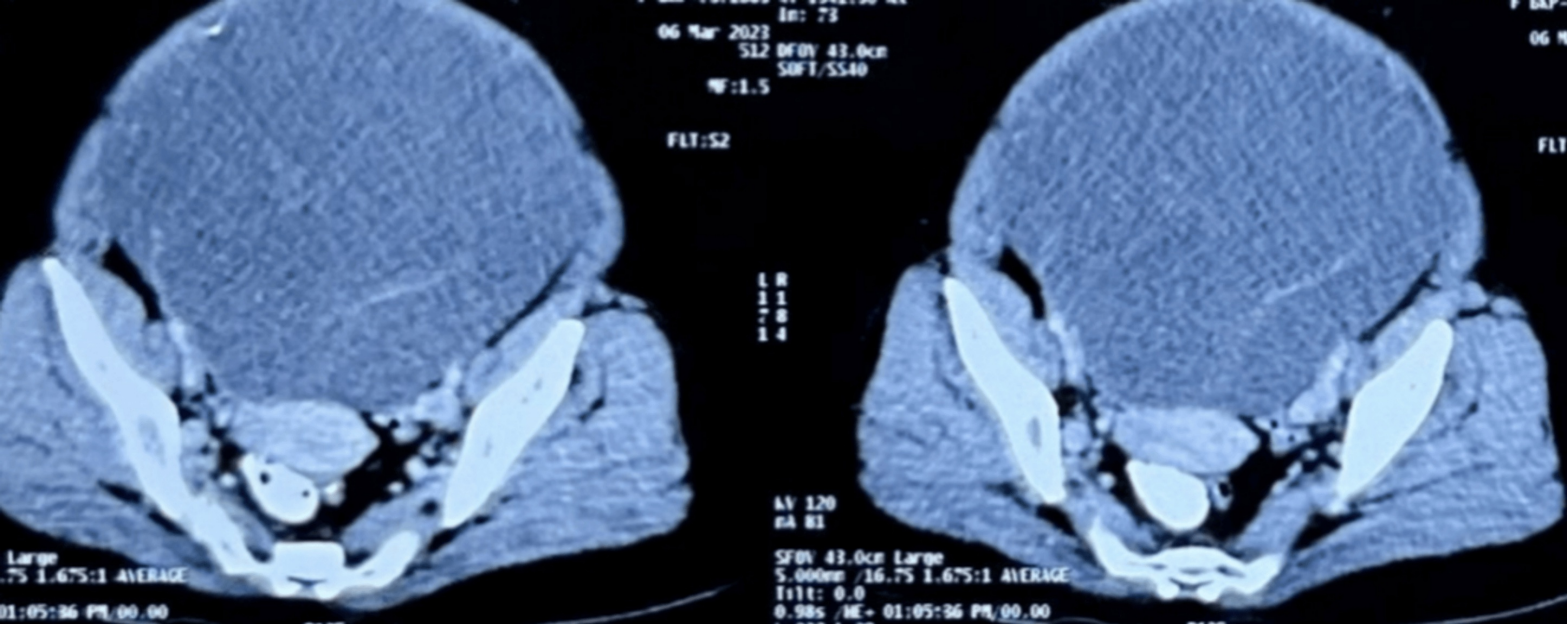
Axial sections of Contrast Enhanced Computed Tomography through the pelvis shows a complex cystic mass occupying the entire pelvis. Ovaries are not identified separately. The cystic mass has thick internal septations with a fatty focus seen in the posterior aspect.

A 52-year-old female came with a history of abdominal distension for four months, which was initially noticed in the lower abdomen and then gradually progressed to the upper abdomen with vague pain and discomfort. On inspection abdomen had grossly distended veins. On palpation a nontender mass measuring 30×20 cm was seen occupying the entire abdomen. Contrast-enhanced computed tomography of abdomen showed a hemorrhagic and a multiseptate cystic lesion extending up to the epigastrium of size 250×150 mm. Ovaries weren't identified separately (Figure 1). Preoperative Ca-125 values were 23.1 U/L (reference range 0-35 U/L) and lactate dehydrogenase values were 186 U/L (reference range 140-280 U/L). Cytology for ascitic fluid showed no malignant cells. A staging laparotomy with total abdominal hysterectomy with bilateral salpingo-oophorectomy along with pelvic lympadenectomy, omentectomy and appendicectomy was performed.

**Figure 2:**
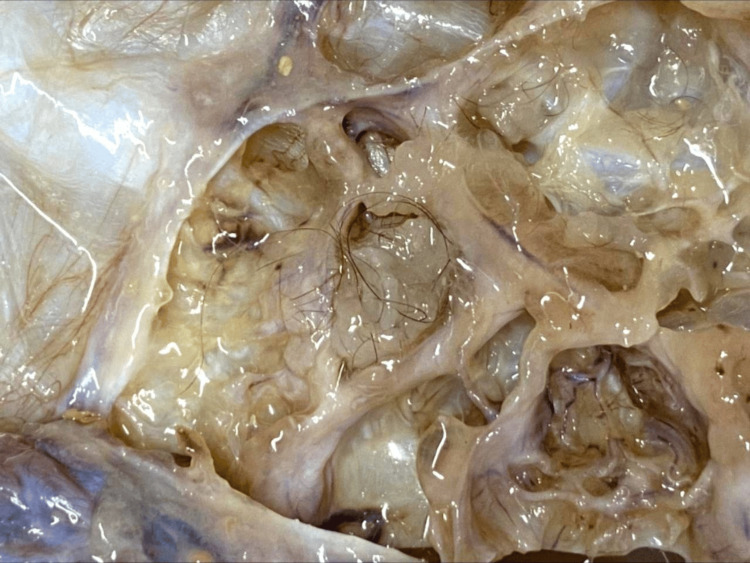
Gross morphology of cystic teratoma shows tufts of hair and multi-loculated cystic areas.

Gross examination of the surgical specimen revealed a globular glistening cystic mass weighing 8.5 kg and measuring 33×30×12 cm. The external surface was grey-white and glistening. Capsular breach was noted adjacent to the right fallopian tube attachment. Cut surface of the cystic mass extruded gelatinous fluid that was brown in colour. The internal surface was multiloculated in appearance, with few cysts extruding cheesy pultaceous material, and hair follicles were noted (Figure 2). The largest cyst measured 15×10×8 cm and the smallest measured 1×1×0.5 cm. Focal areas had papillary excrescences. The uterus, cervix, left ovary and left fallopian tube appeared grossly unremarkable. 

**Figure 3:**
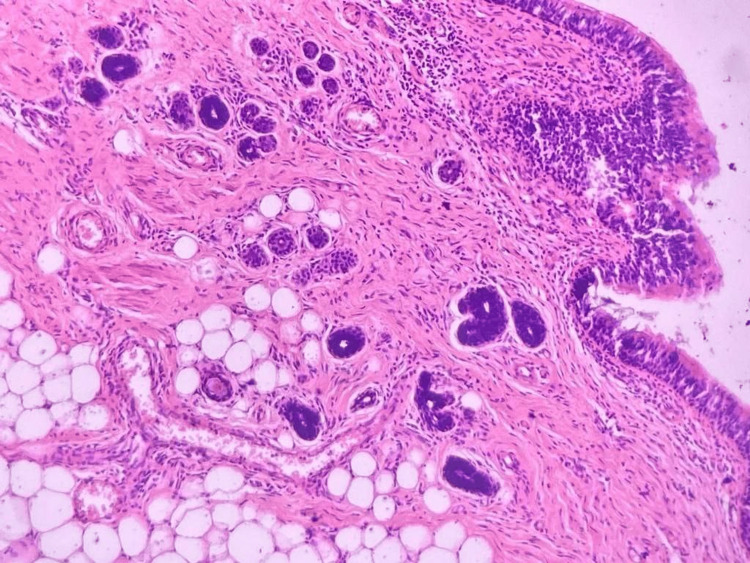
Histopathology shows a cyst with adnexal structures (H&E 400x)

**Figure 4:**
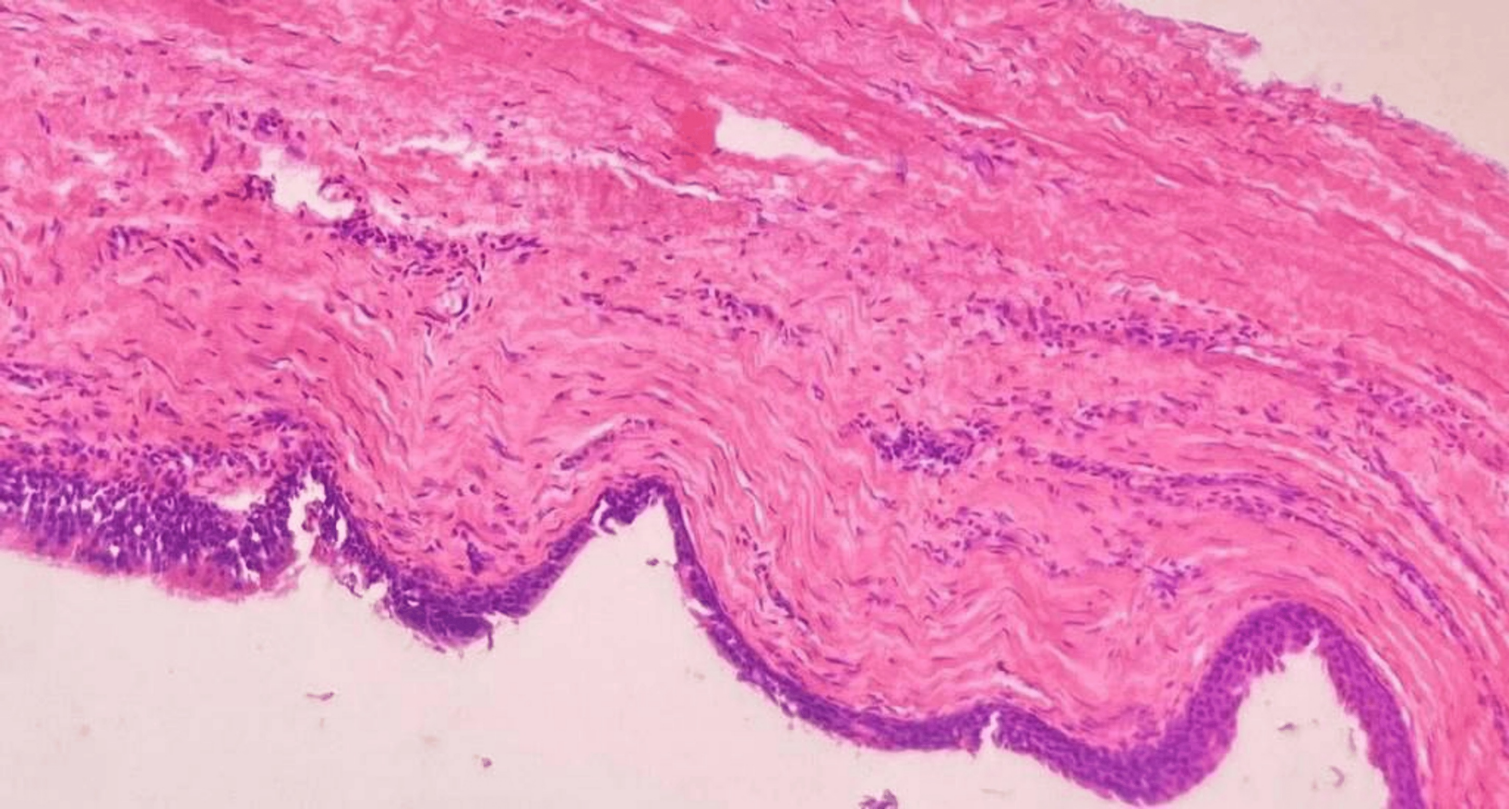
Histopathology shows a cyst wall lined by respiratory epithelium (H&E,400x)

**Figure 5:**
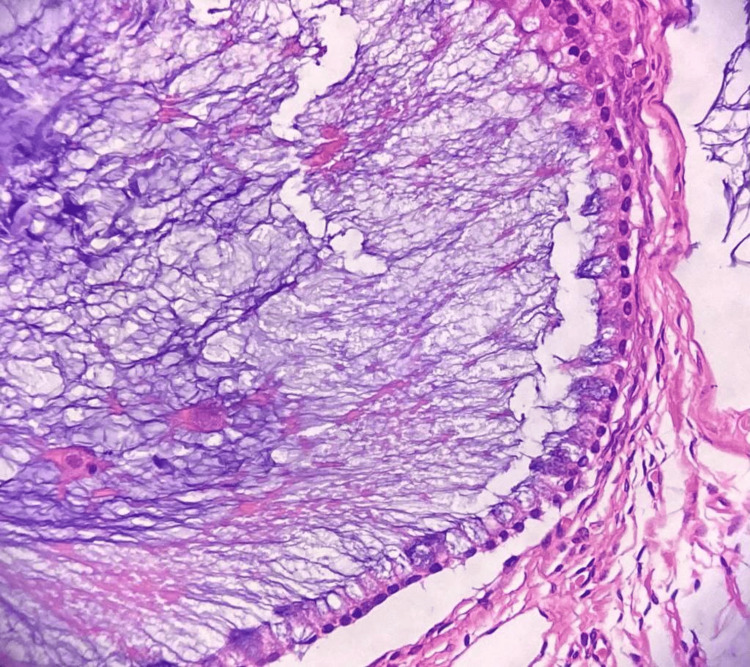
Histopathology shows a pools of mucin with goblet cells (H&E 200x)

**Figure 6:**
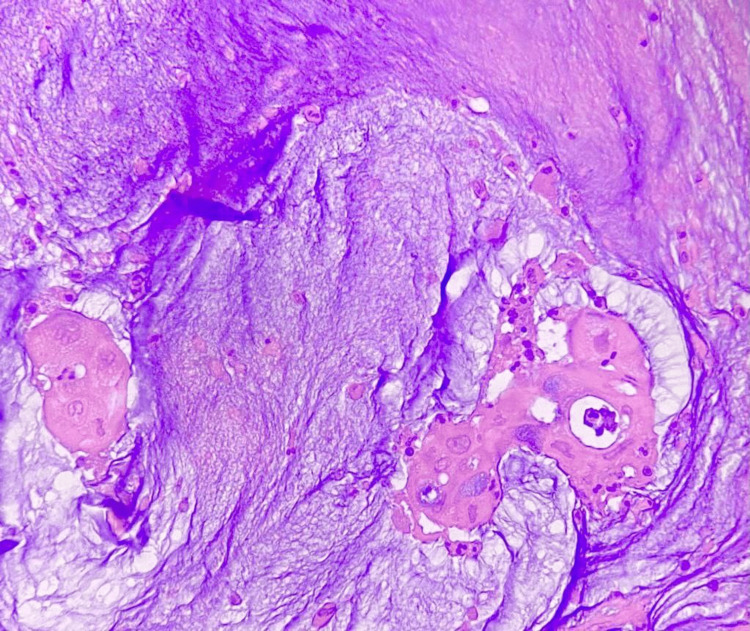
Histopathology shows an adenocarcinoma component in the pool of mucin (H&E 400x)

Histopathological examination of the ovarian mass showed a cystic lesion displaying components derived from all three germ layers, composed of pilosebaceous glands (Figure 3), respiratory epithelium (Figure 4), adipocytes, bony tissue and mucous glands (Figure 5). There were no immature elements, thus indicating features of a mature cystic teratoma. The cyst also showed pools of mucin and was composed of numerous closely packed complex glands. Individual cells showed moderate nuclear pleomorphism, increased N:C ratio, and loss of polarity (Figure 6). The ovarian surface was involved. Histological grade was grade 2 (up to 50% solid foci). The fallopian tube surface was uninvolved. Cervix, uterus, left ovary, bilateral fallopian tube and appendix didn’t show any malignant lesion. Lymph nodes were uninvolved. The final diagnosis was mucinous cystadenocarcinoma arising from the mature cystic teratoma of the right ovary, pT1c2pN0, International Federation of Gynecology and Obstetrics (FIGO) stage: IC2.

## Discussion

Ovarian tumours can be of three types: surface epithelial tumours, sex cord stromal tumours and germ cell tumours. Germ cell tumours account for 20 to 30% of the ovarian neoplasms. Teratomas are germ cell neoplasms that can be benign or malignant. Teratomas contain mature tissues from at least two of the three germ cell layers (ectoderm, mesoderm and endoderm). Studies by Ayahan et al. and Hirakawa et al. found that mature cystic teratoma is a benign tumour, but occasionally undergoes malignant transformation in 1-2% of cases [1,2]. The malignant form is larger than the average mature cystic teratoma. The most common malignancy arising from the teratoma is squamous cell carcinoma [3], while the adenocarcinoma appears only in 6.8% of cases by Guney et al. [4]. Adenocarcinoma arises most frequently from the gastrointestinal or respiratory epithelium. Arising from the respiratory epithelium is very rare. A case report by Boyd et al. also shows adenocarcinoma arising from respiratory epithelium [5].

The most common type of ovarian neoplasm is epithelial tumours, which account for 60 to 65% of all ovarian neoplasms. Mucinous tumours account for 10% of epithelial tumours. Seventy-five percent of mucinous tumours are benign, 20% are borderline and the remaining 5% are malignant. Mucinous tumors can be cystadenomous, borderline or frankly malignant. Mucinous tumours are considered to be the largest of all ovarian tumours. The origin of individual mucinous tumours in the ovary is unclear. Only 5% of mucinous tumours arise from teratomas.

Mature cystic teratomas associated with mucinous cystadenocarcinoma are very rare. It can be due to any of the following hypotheses. It can occur due to collision tumour or due to malignant transformation of intestinal or respiratory epithelium in the teratoma [6-8]. Collision tumour is a neoplastic lesion comprised of two or more distinct neoplasms that develop adjacent to one another and coexist with no or minimal intermingling between them. Various combinations of teratoma with granulosa cell tumour, serous cystadenocarcinoma, steroid cell tumour, neuroblastoma [9], and squamous cell carcinoma have been documented in the past.

Mature cystic teratoma associated with mucinous cystadenocarcinoma usually occurs in post menopausal women. It presents as a rapidly enlarging abdominal mass and other symptoms of advanced malignancy. Malignant transformation is difficult to diagnose preoperatively due to lack of any specific signs and symptoms. Tumour markers are also not helpful in detecting malignant transformation. In the above-mentioned case, the patient presented with abdominal distension and normal tumour markers. CT did not show any evidence of second tumour. Post-operative histopathological examination shows malignant transformation. The ovarian mass depicts an intermediate transitional zone between the benign and malignant components. The management of ovarian carcinoma in general includes a multidisciplinary approach. The surgery to be done is staging laparotomy, which includes extensive sampling from omentum, pelvic and para-aortic lymph nodes, suspicious nodules in any part of peritoneum and hemidiaphragm, along with total abdominal hysterectomy with bilateral salpingo-oophorectomy. Extensive sampling helps in identifying the accurate stage of the disease. Adjuvant treatment required is based on the stage of the disease and grade of the tumour. Since this is a stage Ic2 ovarian tumour, adjuvant chemotherapy of four to six cycles is indicated in this case.

## Conclusions

In conclusion, to the best of our knowledge this is the case of mucinous cystadenocarcinoma arising from the mature cystic teratoma. Although it is a very rare, extensive radiological and histopathological examination is necessary.
